# Valproic acid inhibits interferon-γ production by NK cells and increases susceptibility to *Listeria monocytogenes* infection

**DOI:** 10.1038/s41598-020-74836-w

**Published:** 2020-10-20

**Authors:** Rodolfo Soria-Castro, Alma D. Chávez-Blanco, Blanca Estela García-Pérez, Isabel Wong-Baeza, Raúl Flores-Mejía, Fabián Flores-Borja, Sergio Estrada-Parra, Iris Estrada-García, Jeanet Serafín-López, Rommel Chacón-Salinas

**Affiliations:** 1 Departamento de Inmunología, Escuela Nacional de Ciencias Biológicas, Instituto Politécnico Nacional (ENCB-IPN), Carpio y Plan de Ayala s/n Col. Santo Tomás, C.P. 11340 Mexico City, Mexico; 2 Subdirección de Investigación Básica, Instituto Nacional de Cancerología (INCan), Mexico City, Mexico; 3 Departamento de Microbiología, Escuela Nacional de Ciencias Biológicas, Instituto Politécnico Nacional (ENCB-IPN), Mexico City, Mexico; 4 Laboratorio 103, Sección de Estudios de Posgrado e Investigación, Escuela Superior de Medicina, Instituto Politécnico Nacional (ESM-IPN), Mexico City, Mexico; 5 Centre for Immunobiology and Regenerative Medicine, Barts & The London School of Medicine and Dentistry, Queen Mary University of London, London, UK

**Keywords:** Immunology, Infectious diseases, Bacterial infection

## Abstract

Valproic acid (VPA) is a drug commonly used for epileptic seizure control. Recently, it has been shown that VPA alters the activation of several immune cells, including Natural Killer (NK) cells, which play an important role in the containment of viruses and intracellular bacteria. Although VPA can increase susceptibility to extracellular pathogens, it is unknown whether the suppressor effect of VPA could affect the course of intracellular bacterial infection. This study aimed to evaluate the role of VPA during *Listeria monocytogenes* (L.m) infection, and whether NK cell activation was affected. We found that VPA significantly augmented mortality in L.m infected mice. This effect was associated with increased bacterial load in the spleen, liver, and blood. Concurrently, decreased levels of IFN-γ in serum and lower splenic indexes were observed. Moreover, in vitro analysis showed that VPA treatment decreased the frequency of IFN-γ-producing NK cells within L.m infected splenocytes. Similarly, VPA inhibited the production of IFN-γ by NK cells stimulated with IL-12 and IL-18, which is a crucial system for early IFN-γ production in listeriosis. Finally, VPA decreased the phosphorylation of STAT4, p65, and p38, without affecting the expression of IL-12 and IL-18 receptors. Altogether, our results indicate that VPA increases the susceptibility to *Listeria monocytogenes* infection and suggest that NK cell is one of the main targets of VPA, but further work is needed to ascertain this effect.

## Introduction

Valproic acid (VPA) is a drug approved for the control of neurological disorders including epilepsy, bipolar disorder, anxiety crisis, depression, schizophrenia, post-traumatic stress and migraine^1^. The effects of VPA on these conditions are due to its ability to increase the levels of gamma-aminobutyric acid (GABA), norepinephrine, dopamine and serotonin; and its ability to block voltage-dependent sodium channels^2^. Several clinical trials have also shown that VPA modifies epigenetic regulation and can be used in the control of different types of neoplasms^3^. Through its function as histone deacetylase inhibitor (HDACi), VPA affects the expression of genes involved in DNA repair, cell cycle and apoptosis^4^. Other studies have revealed the anti-inflammatory ability of VPA, by selectively decreasing the production of cytokines in different models of acute or chronic exacerbated inflammation^5^. In fact, VPA can suppress various functions of innate and adaptive immune cells, such as macrophages^6^, mast cells^7^, dendritic cells (DC)^8^, neutrophils^9^, natural killer (NK) cells^10^, γδ T cells^11^, B cells^12^, cytotoxic T cells^13^, and T helper cells^14^. The selective anti-inflammatory effects of VPA, suggest this compound could be exploited over some non-steroidal anti-inflammatory drugs (NSAIDs), which have adverse cardiovascular effects or induce nephrotoxicity and gastrointestinal bleeding^15^.

NK cells belong to the innate immune system and through their cytotoxic activity and ability to secrete significant amounts of IFN-γ they play a central role in the defense against intracellular pathogens and in the control of tumors^16^. IFN-γ activates macrophages and DC, promoting the increased expression of MHC molecules, co-stimulatory molecules, Reactive Oxygen Species (ROS), Nitric Oxide (NO) and inducing autophagy, which is crucial for containment of several intracellular pathogens^17^. IFN-γ production by NK cells is tightly regulated by IL-12 and IL-18, which are usually produced by phagocytic cells after infection with intracellular pathogens^18,19^.

Listeriosis is a foodborne disease caused by *Listeria monocytogenes* (L.m), an intracellular Gram-positive bacteria^20^. Early control of L.m infection relies on IFN-γ production^21,22^. Although IFN-γ is produced by several cell types during L.m infection, they vary throughout the course of infection^23^. In this regard, previous studies have shown that NK cells are a major source of this cytokine in the early stages of listeriosis^24,25^. In vitro studies have shown that IFN-γ production by NK cells depends on IL-12 and IL-18 released by L.m-infected phagocytic cells^18,26^. Remarkably, previous reports have shown that VPA interferes with NK cell activation, decreasing their cytotoxic capacity and in vitro IFN-γ production in response to IL-12, IL-15 and IL-18^10,27^. Therefore, in this study, we analyzed whether VPA increased the susceptibility to L.m infection in vivo and whether VPA altered in vitro IFN-γ production by NK cell in a mouse model of L.m infection.

## Results

### Valproic acid increases the susceptibility to Listeria monocytogenes in vivo

**Figure 1:**
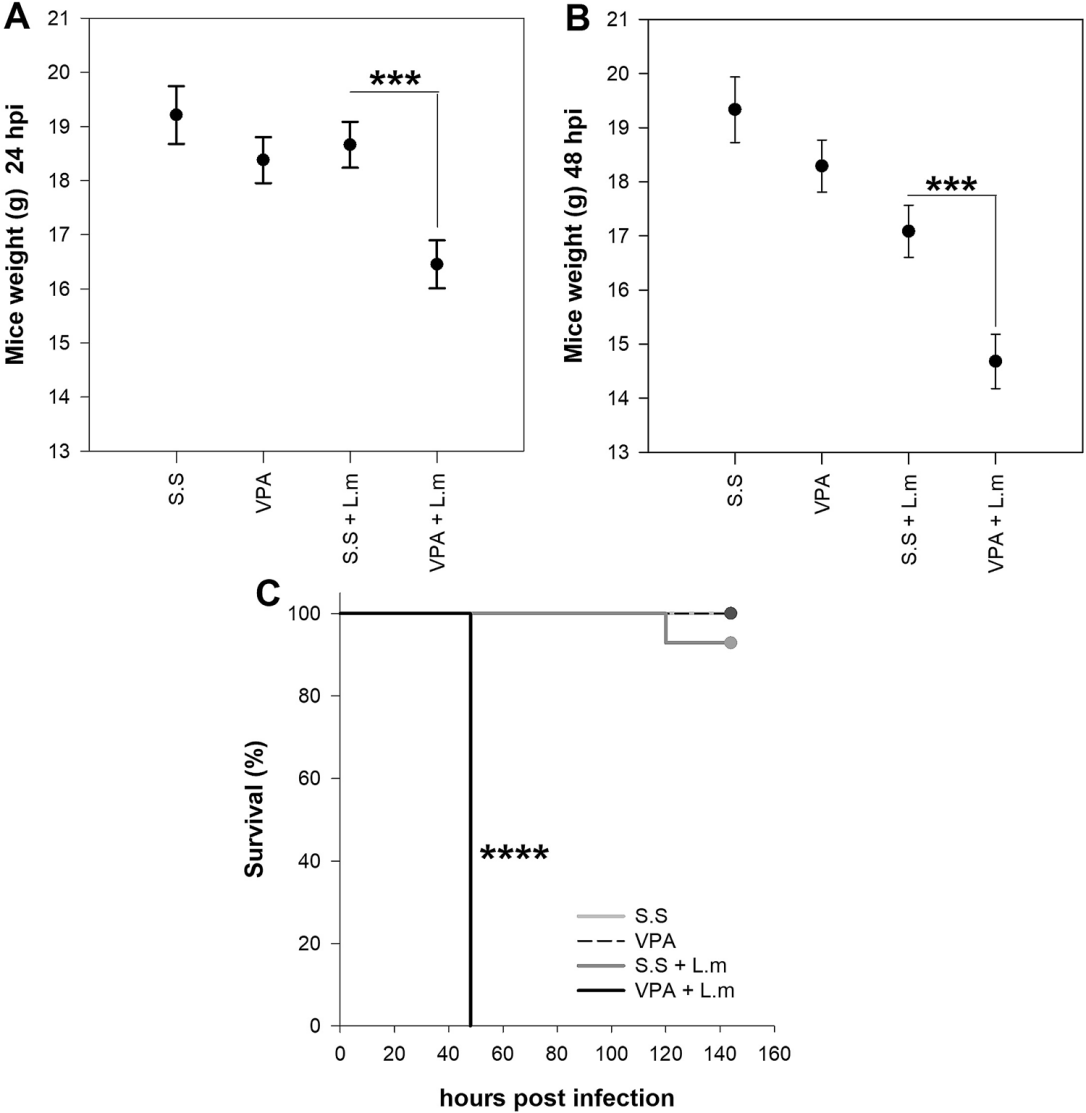
Valproic acid increases susceptibility to in vivo infection by *Listeria monocytogenes*. BALB/c mice were treated with VPA (500 mg/Kg) or s.s 15 min before infection with 2.5 × 10^5^ CFU L.m i.p. (**A**) mice weight (g) at 24 and (**B**) mice weight at 48 hpi (***p < 0.001, VPA + L.m vs s.s + L.m groups at 24 and 48 hpi). Data are expressed as adjusted means in grams (g) with 95% confidence intervals; one way-ANCOVA. (**C**) Kaplan–Meier mouse survival curves after L.m infection (****p < 0.0001, VPA + L.m vs s.s + L.m groups). n = 9–14 per group.

**Figure 2:**
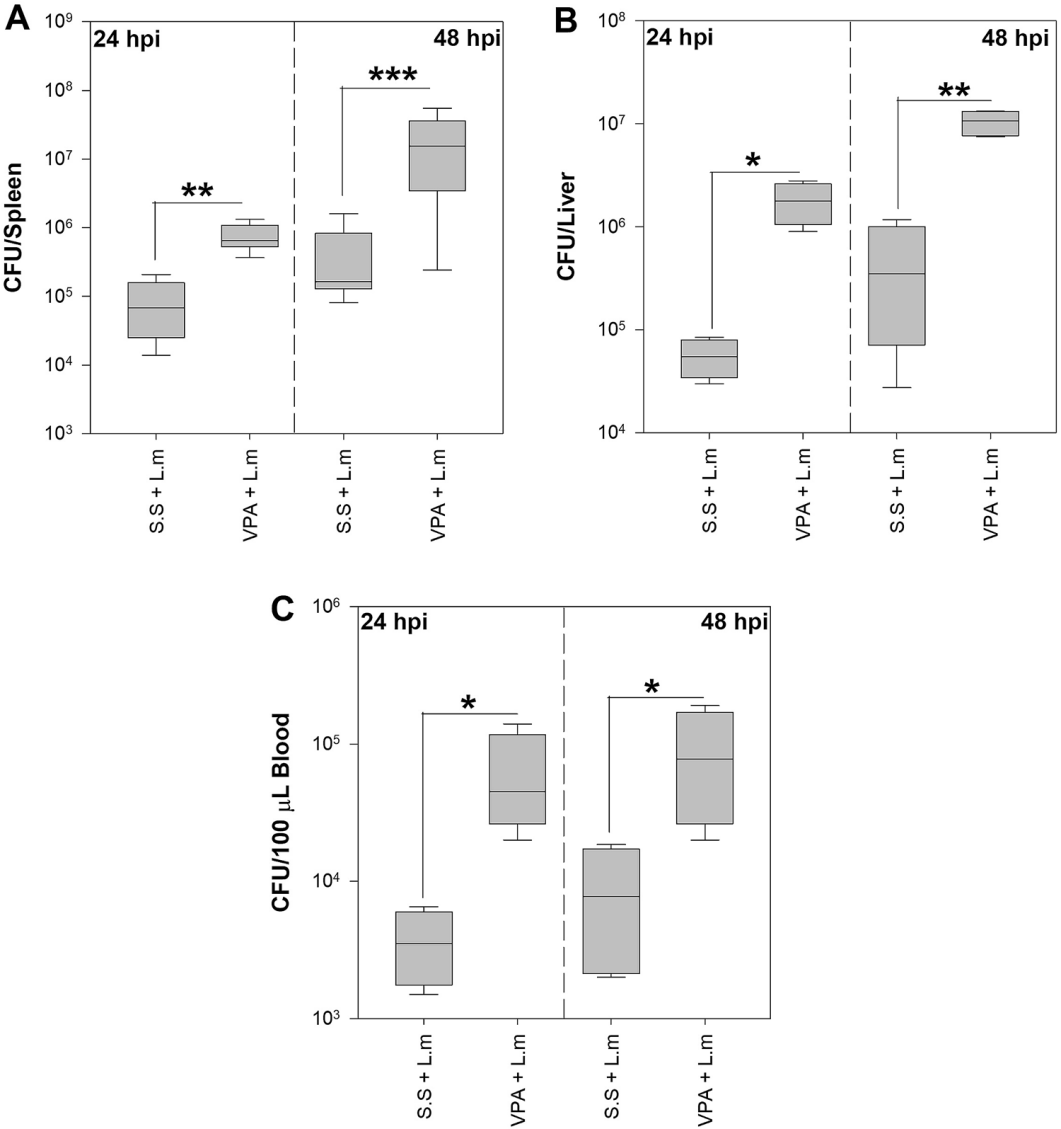
Valproic acid increases the bacterial load in the spleen, liver and blood of mice infected with *Listeria monocytogenes*. Bacterial load in the (**A**) Spleen of BALB/c mice infected with 2.5 × 10^5^ CFU L.m following treatment with VPA (500 mg/Kg) or s.s (n = 11 per group; **p < 0.01 at 24 hpi and ***p < 0.001 at 48 hpi). (**B**) Liver (n = 4 per group; *p < 0.05 at 24 hpi and **p < 0.01 at 48 hpi) and (**C**) Blood (n = 4 per group; *p < 0.05 at 24 and 48 hpi). Data are expressed as median and range; Kruskal–Wallis.

VPA can dampen the immune response to several pathogens by interfering or hindering the activation of relevant immune cells^5^. To determine if these effects are observed during murine listeriosis, BALB/c mice were inoculated with VPA 15 min before infection with L.m. We observed that VPA-treated mice have a more pronounced decrease in weight at 24 and 48 h post-infection (hpi) (*p* < 0.001) (Fig. 1A,B) and increased mortality to L.m infection when compared with those infected mice that received saline solution (s.s.) (*p* < 0.0001) (Fig. 1C). Furthermore, VPA-treated mice showed increased bacterial load 24 and 48 hpi in the spleen (*p* < 0.01, 24 hpi; *p* < 0.001, 48 hpi; Fig. 2A), liver (*p* < 0.05, 24 hpi; *p* < 0.01, 48 hpi; Fig. 2B) and blood (*p* < 0.05, 24 and 48 hpi; Fig. 2C) in relation to infected mice that received s.s.

**Figure 3:**
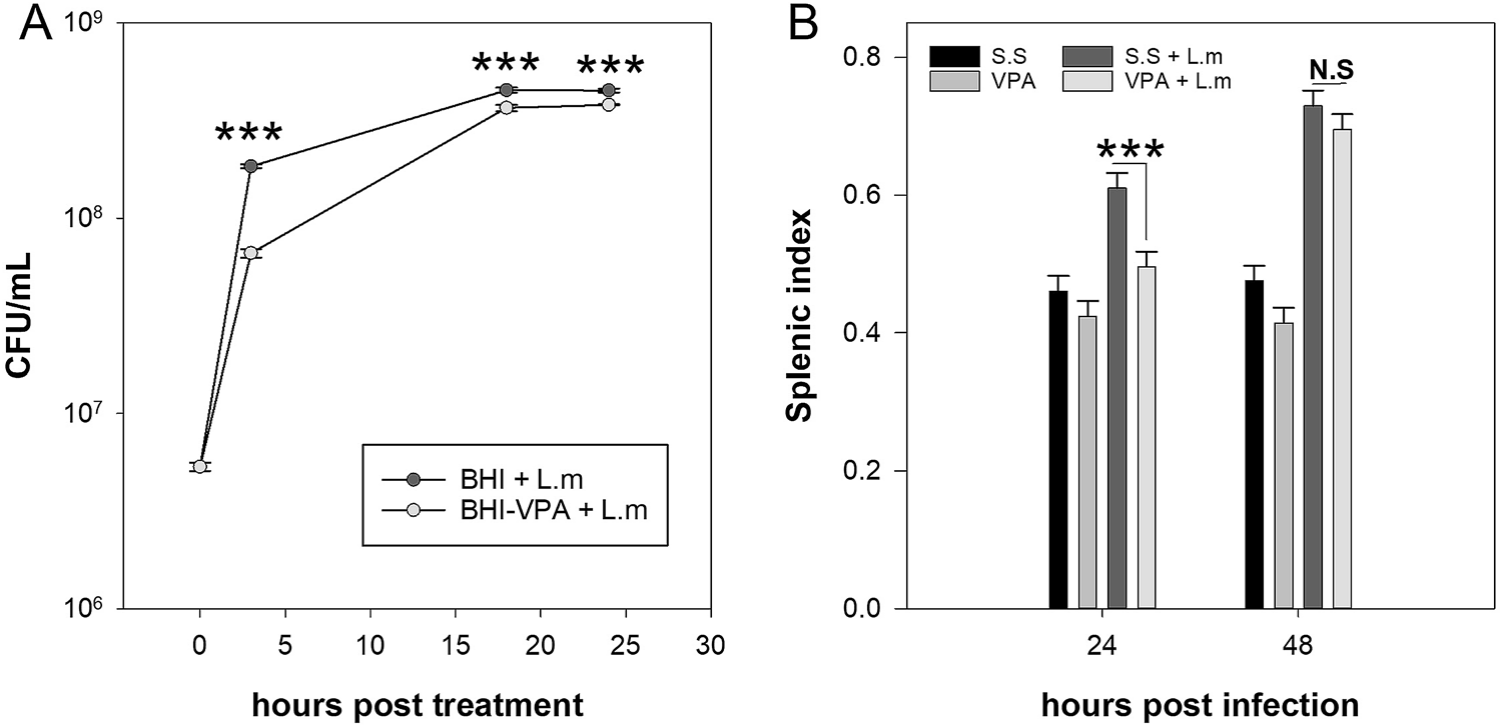
Valproic acid inhibits early splenomegaly during *Listeria monocytogenes* infection. (**A**) L.m were cultured in BHI broth in presence or absence of 52 mM VPA and bacterial viability was evaluated at different times by CFU assay (n = 3 per group; ***p < 0.001). Data are expressed as mean ± s.e.m; two way-RM ANOVA (**B**) BALB/c mice were treated with VPA (500 mg/Kg) or s.s 15 min before infection with 2.5 × 10^5^ CFU L.m. 24 and 48 hpi the splenic index was evaluated (n = 15 per group; ***p < 0.001, VPA + L.m vs s.s + L.m groups at 24 h; N.S., Not Significant). Data are expressed as mean ± s.e.m; two-way ANOVA.

Increased bacterial load in mice could be the result of VPA directly promoting bacterial replication or through alterations in the host immune response. To discern between these two possibilities, L.m was grown in BHI broth in presence or absence of VPA, and at different times bacterial growth was evaluated through Colony Formation Unit (CFU) assay. We observed that VPA did not promote bacterial growth in vitro at any time point evaluated, but instead inhibited bacterial growth (*p* < 0.001) (Fig. 3A). Next, we evaluated if splenomegaly, an indicator of immune system activation in L.m infection^28^, was affected by VPA. We noticed that 24 hpi VPA-treated mice showed a decreased splenic index in comparison with infected mice that received s.s (*p* < 0.001) (Fig. 3B), suggesting that VPA hinders the host immune response.

**Figure 4:**
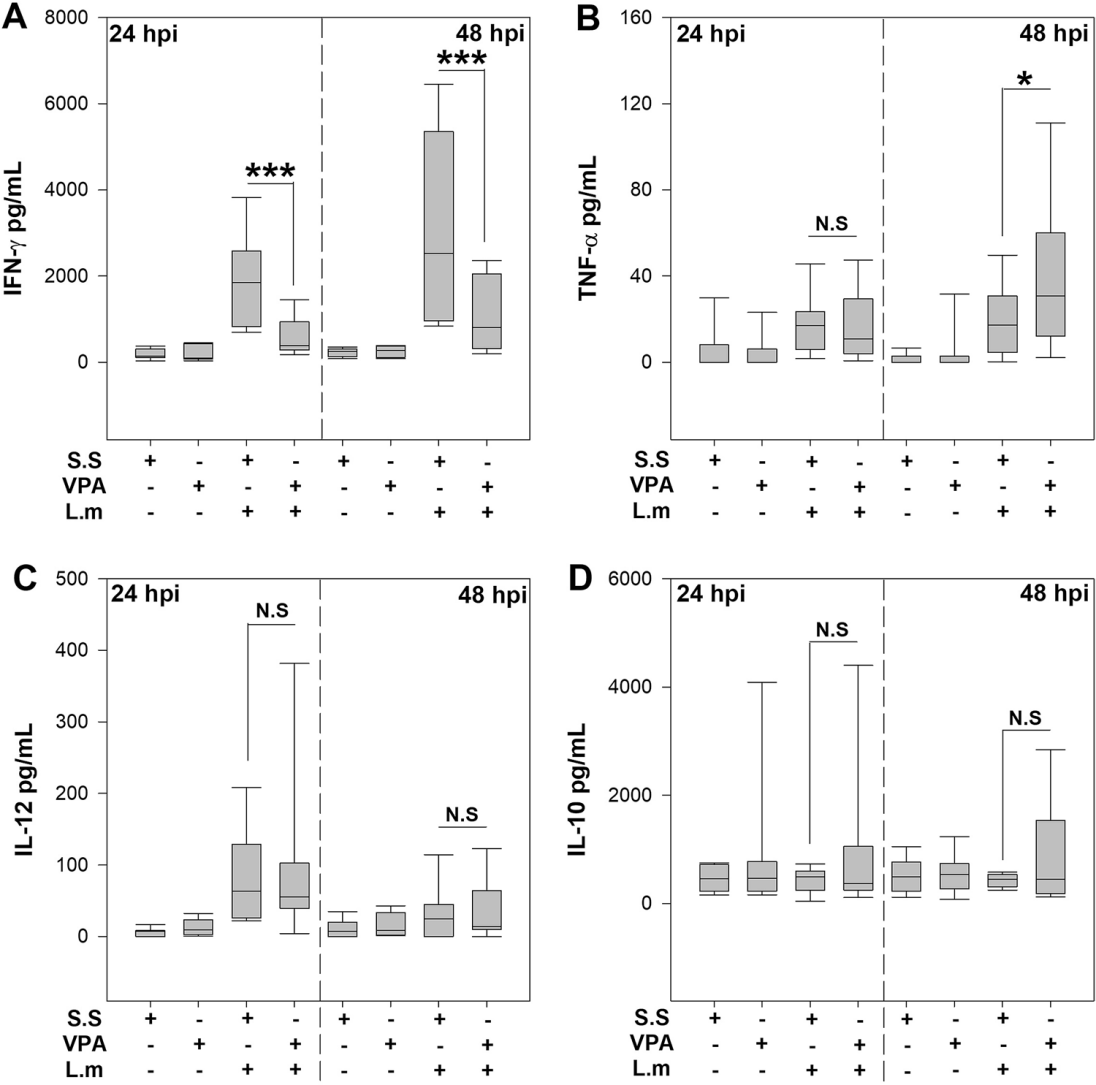
Valproic acid decreases IFN-γ serum levels during *Listeria monocytogenes* infection in vivo. BALB/c mice were treated with VPA (500 mg/Kg) or s.s 15 min before infection with 2.5 × 10^5^ CFU L.m i.p. 24 and 48 hpi the cytokines (**A**) IFN-γ (***P < 0.001, VPA + L.m vs s.s + L.m groups at 24 and 48 hpi), (**B**) TNF-α (*P < 0.05, VPA + L.m vs s.s + L.m groups at 48 hpi), (**C**) IL-12p70 and (**D**) IL-10 were evaluated in serum by ELISA. (n = 11 per group; N. S., Not Significant). Data are expressed as median and range; Kruskal–Wallis.

Cytokines play a crucial role in the control of L.m infection, and in particular IFN-γ and TNF-α are associated with infection control^23^. Therefore, we investigated whether VPA affects serum levels of IFN-γ and TNF-α in L.m infected mice. Interestingly, we noticed that 24 and 48 hpi VPA induced a significant reduction in IFN-γ levels in serum in comparison with infected mice that received s.s (*p* < 0.001) (Fig. 4A). However, we observed that VPA did not modify TNF-α induced by L.m at 24 hpi but induced an increase at 48 hpi (*p* < 0.05; Fig. 4B). Because IL-12 is crucial for early IFN-γ production in L.m infection^29^, we evaluated whether VPA altered its production. Remarkably, we observed that VPA did not modify IL-12p70 induced by L.m at 24 or 48 hpi (Fig. 4C). On the other hand, IL-10 is a strong inhibitor of IFN-γ production in L.m infection^30^, we next evaluated if VPA treatment favored IL-10 production during L.m infection. We noticed that L.m infection was unable to induce IL-10 production at 24 or 48 hpi, and VPA did not affect IL-10 at these time points (Fig. 4D). These findings indicate a cytokine-specific effect of VPA compromising early IFN-γ production, that is associated with a poor bacterial control and increased susceptibility to L.m infection in BALB/c mice.

### Valproic acid inhibits IFN-γ production by NK cells during in vitro infection with Listeria monocytogenes

**Figure 5:**
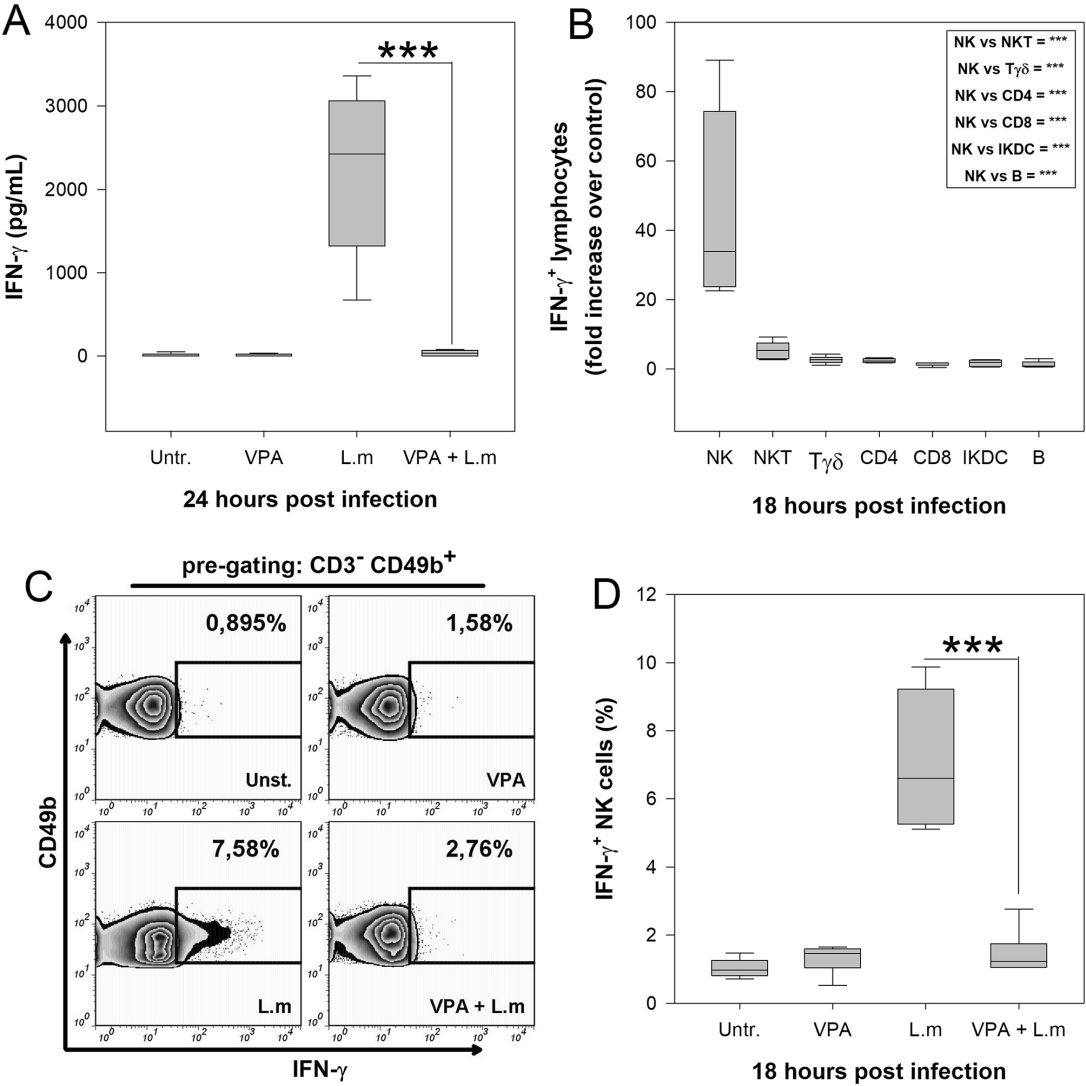
Valproic acid inhibits IFN-γ-producing NK cells during *Listeria monocytogenes* infection in vitro. 1 × 10^6^ Splenocytes from healthy BALB/c mice were pre-incubated 1 h with VPA and then co-cultured with L.m at a MOI of 0.1:1. (**A**) IFN-γ level in cell supernatants was evaluated by ELISA 24 hpi n = 9 per group; ***p < 0.001 VPA + L.m vs L.m groups; Untr., Untreated cells). (**B**) Splenocytes were infected with L.m and 18 hpi cells were stained and analyzed by flow cytometry. Graph depicts the fold increase in numbers of IFN-γ producing cells upon infection with L.m, compared to uninfected cultures. The box shows the statistical significance between different immune cell populations compared to NK cells. (n = 6 per group). (**C**) Representative flow cytometry zebra-plots showing the frequency of CD3-CD49b + IFN-γ producing NK cells. Splenocytes were left uninfected, treated with VPA only or infected with L.m in presence or absence of VPA. (**D**) Percentage of IFN-γ + NK cells. (n = 6 per group; ***p < 0.001 between VPA + L.m vs L.m groups; Untr., Untreated cells). Data are expressed as median and range; Kruskal–Wallis.

Mouse spleen is a target organ of L.m infection and several spleen lymphoid populations have been identified to produce IFN-γ in the early phase of infection^24,25^. We next determined if VPA could affect splenocyte production of IFN-γ induced by L.m in vitro. To this end, BALB/c splenocytes were treated with 2 mM VPA, a concentration that does not affect cellular viability (Fig. S1), for 1 h and infected with 0.1 MOI L.m during 24 h. We noticed that VPA inhibited IFN-γ production induced by L.m infection in relation to splenocytes stimulated only with the bacterium (*p* < 0.001) (Fig. 5A).

To identify the main cell population involved in IFN-γ production after L.m infection, first we evaluated by flow cytometry the time at which the maximum percentage of IFN-γ-producing cells were induced. We found that 18 hpi, the highest percentage of IFN-γ-producing cells was observed (Fig. S2). Next, we determined by flow cytometry which cell populations produced IFN-γ at this point (Fig. S3). We observed that within splenocytes infected with L.m*,* NK, NKT, Tγδ, T CD4 + , but not CD8 + , IKDC or B cells induced IFN-γ production (Table S1).

Furthermore, among lymphoid cells, NK cells were the main population that produced IFN-γ (Fig. 5B). Interestingly, VPA treatment significantly ablated IFN-γ producing NK cells induced by L.m infection in vitro (*p* < 0.001) (Fig. 5C,D). These results demonstrate that VPA negatively impacts the production of IFN-γ by NK cells upon in vitro infection with L.m*.*

### Valproic acid reduces IFN-γ production by NK cells in response to IL-12 and IL-18

**Figure 6:**
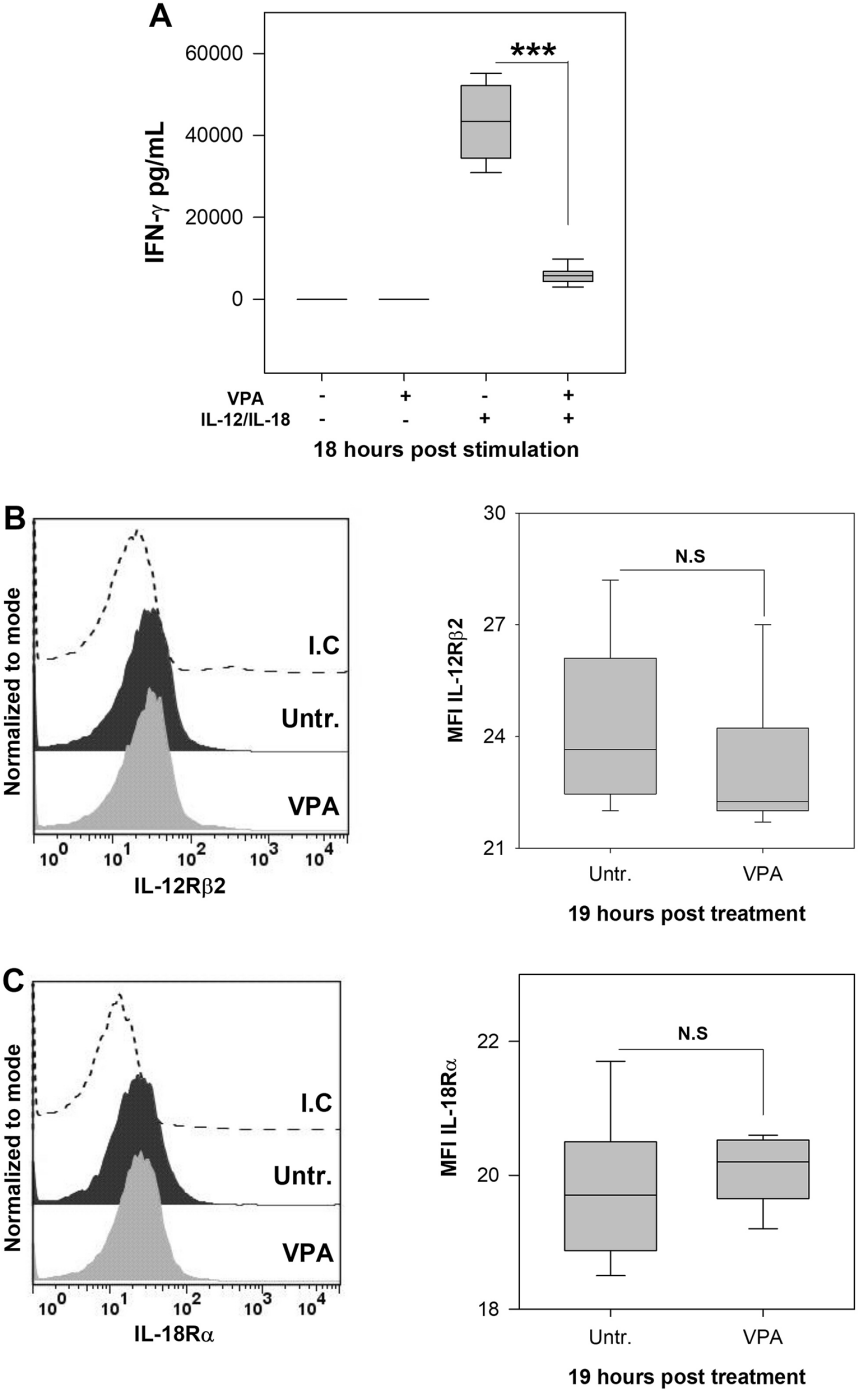
Valproic acid decreases IFN-γ production by NK cells induced by IL-12 and IL-18. (**A**) 5 × 10^4^ purified spleen NK cells from were pre-incubated 1 h with VPA (2 mM) and then stimulated with IL-12 (20 ng/mL) plus IL-18 (100 ng/mL) for 18 h. IFN-γ levels were evaluated in culture supernatants by ELISA (n = 10 per group; ***p < 0.001, VPA + IL-12/IL-18 vs IL-12/IL-18 groups). NK cells were treated or not with VPA for 19 h and the expression of (**B**) IL-12Rβ2 and (**C**) IL-18Ra was evaluated by flow cytometry. The histograms show representative examples of IL-12Rβ2 and IL-18Ra expression in untreated or VPA-treated NK cell. Dotted line represents an isotype control (I.C.). The graphs show the mean fluorescence intensity (MFI). (n = 6 per group; N.S., Not Significant; Untr., Untreated cells). Data are expressed as median and range; Mann Whitney or Kruskal–Wallis, as appropriate.

IL-12 and IL-18 are produced during infectious processes and both play a crucial role in early IFN-γ production^31,32^. During in vitro infection with L.m, IL-12 and IL-18 are secreted by infected phagocytic cells, which then activate IFN-γ production by NK cells^18,26^. Previous studies have shown that VPA decreases IFN-γ production of human and C57BL/6 mouse NK cells treated with IL-12, IL-15 and IL-18, alone or in combination^10,27^. Therefore, we evaluated whether VPA affected IFN-γ production by splenic NK cells from BALB/c mice upon stimulation with IL-12 plus IL-18. We observed that purified NK cells treated for 1 h with 2 mM VPA drastically reduced IFN-γ production upon stimulation with IL-12 plus IL-18 (*p* < 0.001) (Fig. 6A). As VPA can affect the expression of activating receptors on the membrane of several immune cells^5^, we evaluated whether VPA affected the expression of IL-12 and IL-18 receptors on NK cells. We noticed that VPA did not induce a significant reduction in the expression of both receptors on NK cells (Fig. 6B,C). These results indicate that the decreased production of IFN-γ by NK cells stimulated with IL-12 plus IL-18 does not depend on variations in their receptors expression level.

**Figure 7:**
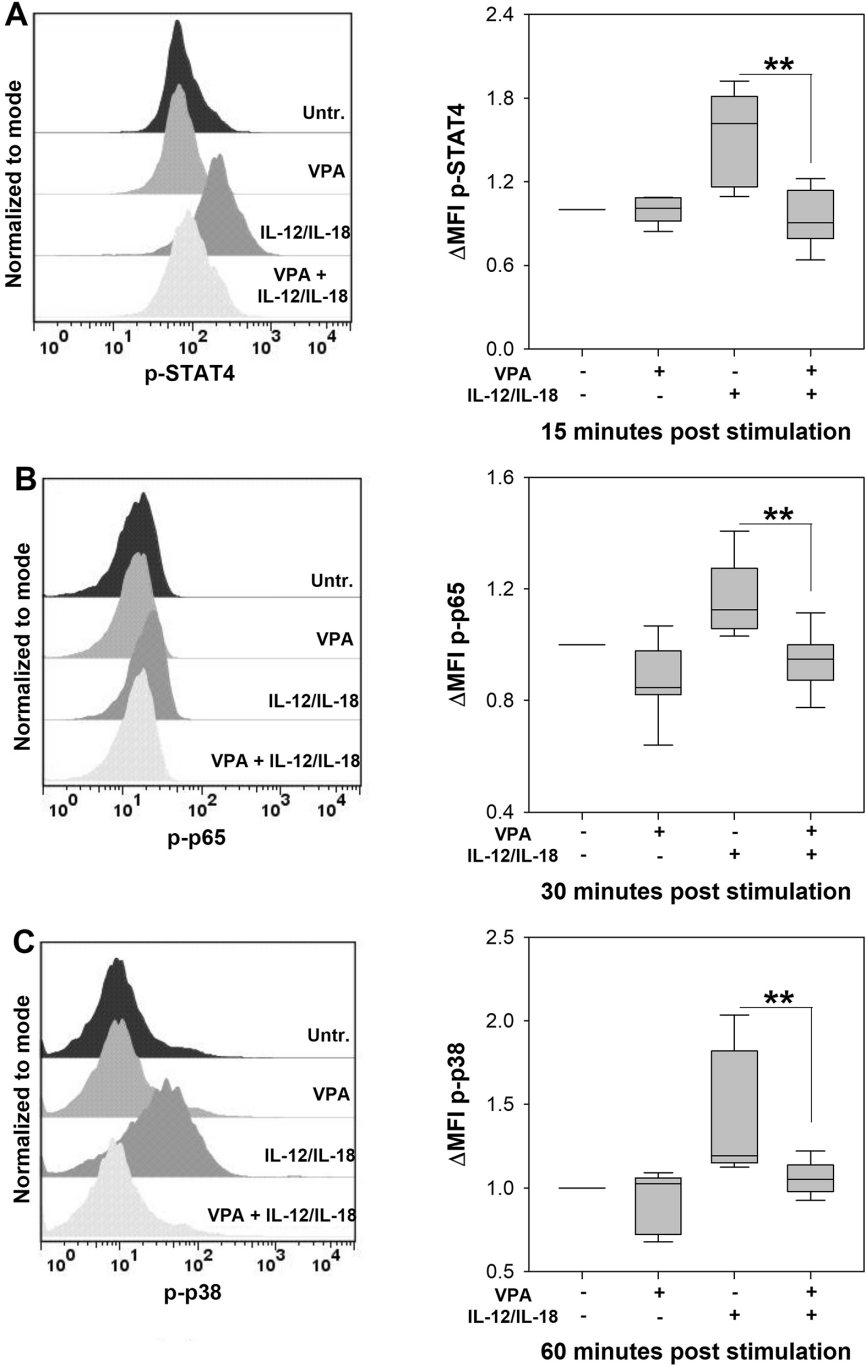
Valproic acid affects cell signaling induced by IL-12 and IL-18 in NK cells. 5 × 10^4^ splenic NK cells from BALB/c mice were cultured with VPA for 1 h and then stimulated with IL-12 plus IL-18 for different times to determine the phosphorylation of (**A**) STAT4 at 15 min, (**B**) p65 at 30 min and (**C**) p38 at 60 min by flow cytometry. The histograms show representative examples of levels of phosphorylated STAT4, p65 and p38 under the different stimulation conditions. The graphs show the fold change in mean fluorescence intensity (ΔMFI) compared to unstimulated NK cells. (n = 7–8 per group; **p < 0.01 (STAT4), **p < 0.01 (p65) and **p < 0.01 (p38), VPA + IL-12/IL-18 vs IL-12/IL-18 groups; Untr., Untreated cells). Data are expressed as median and range; Kruskal–Wallis.

A second mechanism that VPA employs to inhibit immune cell activation is through altering the cell signaling pathways of different activating receptors in immune cells^5^. Here we evaluated whether the activation of key cell signaling molecules induced by IL-12 and IL-18^19,33^ was inhibited by VPA. NK cells treated with VPA showed lower levels of phosphorylated -STAT4 (*p* < 0.01) (Fig. 7A), -p65 (*p* < 0.01) (Fig. 7B) and -p38 (*p* < 0.01) (Fig. 7C) upon stimulation with IL-12 and IL-18. These results indicate that VPA inhibits IFN-γ production by NK cells through suppressing the activation of critical cell signaling proteins in the IL-12R and IL-18R pathways.

Altogether, these results show that VPA increases the susceptibility to L.m infection by decreasing IFN-γ production. Our in vitro findings suggest that this effect may be explained by an impairment of the IL-12 and IL-18 signaling pathways required for IFN-γ production by NK cells.

## Discussion

NK cells play a crucial role in the innate immune response to tumors and different types of pathogens, including bacteria^16^. Two main effector functions are associated to this purpose: granule mediated cytotoxicity and the production of different cytokines and chemokines that maintain inflammation^34^. In particular, NK cell-derived IFN-γ plays a crucial role for the modulation of macrophages, dendritic cells and T cell polarization toward a Th1 profile, which contribute to pathogen control^35^.

VPA exerts modulatory effects on several immune cells, including NK cell activation by IL-12, IL-15 and IL-18^10,27^, which is crucial step in the early immune response to pathogens^19^. In this study, we demonstrate that VPA affects the susceptibility of BALB/c mice to L.m infection, by interfering with key elements of the immune response, such as IFN-γ production which then favors an increased bacterial load in target organs.

This result is in agreement with the role of VPA in other in vivo models of infectious diseases like *Klebsiella pneumoniae* and *Candida albicans*, where the innate immune response mediated by phagocytic cells was altered^8^. Interestingly, in vitro models of infections have reported contrasting results. For instance, VPA treatment results in higher replication in macrophages of *Staphylococcus aureus* and *Escherichia coli*^6^, while in *Mycobacterium tuberculosis* infection VPA inhibits intracellular growth in macrophages^36^.

Although macrophages are central in the immune response to L.m, we focused our study in the analysis of IFN-γ production, because this cytokine plays a critical role in the early control of infection^21,22^, it was severely affected by VPA and the diminished IFN-γ production was associated with poor control in bacterial load.

Interestingly, in our study, VPA effect was cytokine specific as IFN-γ decrease in mice sera was not associated with decreased expression of cytokines that regulate IFN-γ production, such as IL-12, TNF-α and IL-10^23,30^. The response to VPA varies in other inflammation models, for instance, it promotes an anti-inflammatory environment mediated by increased IL-10 production in a model of ischemia/reperfusion injury^37^; reduces serum IL-12p40 levels in the Pam3csk4-induced toxic shock model^8^; and strongly decreases TNF-α levels in bronchial-alveolar lavage in a LPS-induced acute lung damage model^38^. Contrasting with these results, VPA is unable to decrease serum TNF-α levels in the CLP-induced septic encephalopathy model^39^. These studies suggest that VPA modulates cytokine environment in a specific manner depending on the activation signals.

Several innate immune cells have been identified to produce IFN-γ during L.m infection^23^. Here we noticed that NK cells were the main producers when spleen cells were infected with L.m in vitro, as it has been previously described in C57BL/6 mice^24,25^. Remarkably, in this in vitro system VPA reduced the percentage of IFN-γ-producing NK cells suggesting they are the target of this drug during L.m infection. However, it must be remarked that several studies have shown that NK cells can be detrimental to the control of L.m infection^40–42^. NK cells are only one of many cell populations producing IFN-γ at early times, although this effect wanes at later times^42^, and other immune cell populations are equally or more relevant than NK cells in maintaining IFN-γ production in vivo at later stages of infection^43,44^. The relevance of the observed in vitro effect of VPA on NK cells needs to be further characterized in vivo, in order to discern whether it could affect other IFN-γ producing cells during L.m infection.

IFN-γ production by NK cells relies on signals by IL-12 and IL-18 produced by phagocytic cells infected with L.m^18,26^. VPA was able to block IFN-γ release by NK cells when activated with IL-12 and IL-18 without affecting their receptor expression. This result diverges with the effect of Trichostatin A (TSA), a different HDACi, that inhibits the transcription of IL-2, IL-12, IL-15 and IL-18 receptors in NK cells^27^. This dissimilarity could be explained by differences in the specificity and affinity for HDACs between VPA and TSA^45^.

Because cytokine receptors were not affected by VPA, we evaluated whether cell signaling was affected. IL-12 classical signaling pathway promotes the phosphorylation and dimerization of STAT4, while IL-18 promotes NF-κB activation and the MAPK pathway^19^. Furthermore, the synergistic effect of IL-12 and IL-18 in IFN-γ production is due to p38 phosphorylation, which stabilizes IFN-γ mRNA^33^. Interestingly, we found that VPA decreased the phosphorylation of p65 (NF-κB), STAT4, and p38. To the best of our knowledge, this is the first report that shows that STAT4 activation is targeted by VPA. Moreover, a recent study showed that STAT4-deficient mice are more susceptible to L.m infection, with a decreased IFN-γ production and an increased bacterial load^46^. Whether VPA alters STAT4 activation in NK cells or other IFN-γ producing cells during L.m infection in vivo needs to be explored.

Previously, Alvarez et al. showed that VPA inhibited STAT5 phosphorylation in NK cells stimulated with IL-12, IL-15 e IL-18^10^. STAT5 plays a crucial role in cell signaling of IL-15 receptor^47^. How VPA inhibits STAT activation is unknown. One possibility is that VPA, through its HDACi activity, alters STAT acetylation which is tightly regulated by histone acetyl transferases, histone deacetylases and sirtuins. Interestingly, STAT acetylation plays a crucial role for STAT activation and phosphorylation^48^. Whether STAT4 activation is regulated by acetylation needs to be further analyzed.

Several reports have shown the effect of HDCAi on NF-κB activity, for instance: VPA has an inhibitory effect on NF-κB activation in human NK cells stimulated with IL-2^49^; and TSA reduced NF-κB translocation to the nucleus in NK cells stimulated with IL-12, IL-15 and IL-18^27^. Furthermore, VPA is able to inhibit NF-κB activation in other cell populations, like monocytes^50^, macrophages^51^, dendritic cells^52^, fibroblast^53^ and epithelial cells^54^.

Interestingly, the effect of VPA on p38 activation is controversial. Previous work on RAW 264.7 macrophages showed that VPA decreases p38 phosphorylation^55^, while in other reports, VPA activated p38 in microglia cells^56^, brain endothelial cells^57^, and retinal pigment epithelial cells^58^. These studies indicate that VPA modulates the activation of p38 depending on the cell lineage.

Our results suggest that VPA could be used to target NK cells in pathological injury. In fact, VPA has shown promising effects in down-modulating inflammation in different pathological models such as during intestinal injury, autoimmune encephalomyelitis and acute allograft rejection^14,59,60^. Interestingly, NK cells are associated with a number of inflammatory pathologies such as type1 diabetes^61^, psoriasis^62^, lichen planus^63^, rheumatoid arthritis^64^, periodontal disease^65^, chronic obstructive pulmonary disease^66^, atherosclerosis^67^, among others. The main pathological mechanism associated with the aforementioned inflammatory conditions is an exacerbated IFN-γ production by NK cells. Further studies are required to ascertain whether VPA could be used as a therapeutic drug to target NK cell activation and treat those pathologies.

Despite the potential therapeutic use of VPA as an anti-inflammatory agent is quite attractive, it also needs to be considered that VPA treatment could increase the susceptibility of patients to infections with L.m or other infectious agents such as the opportunistic extracellular pathogens *Klebsiella pneumoniae* and *Candida albicans*^8^*.* To the best of our knowledge, an association between the treatment with VPA and an increased incidence of infections has not been described, but further studies are required, in particular in L.m susceptible patients, like infants, elderly, pregnant women, immunocompromised subjects, diabetics, cancer patients, autoimmune diseases patients, and alcoholics^68^.

**Figure 8:**
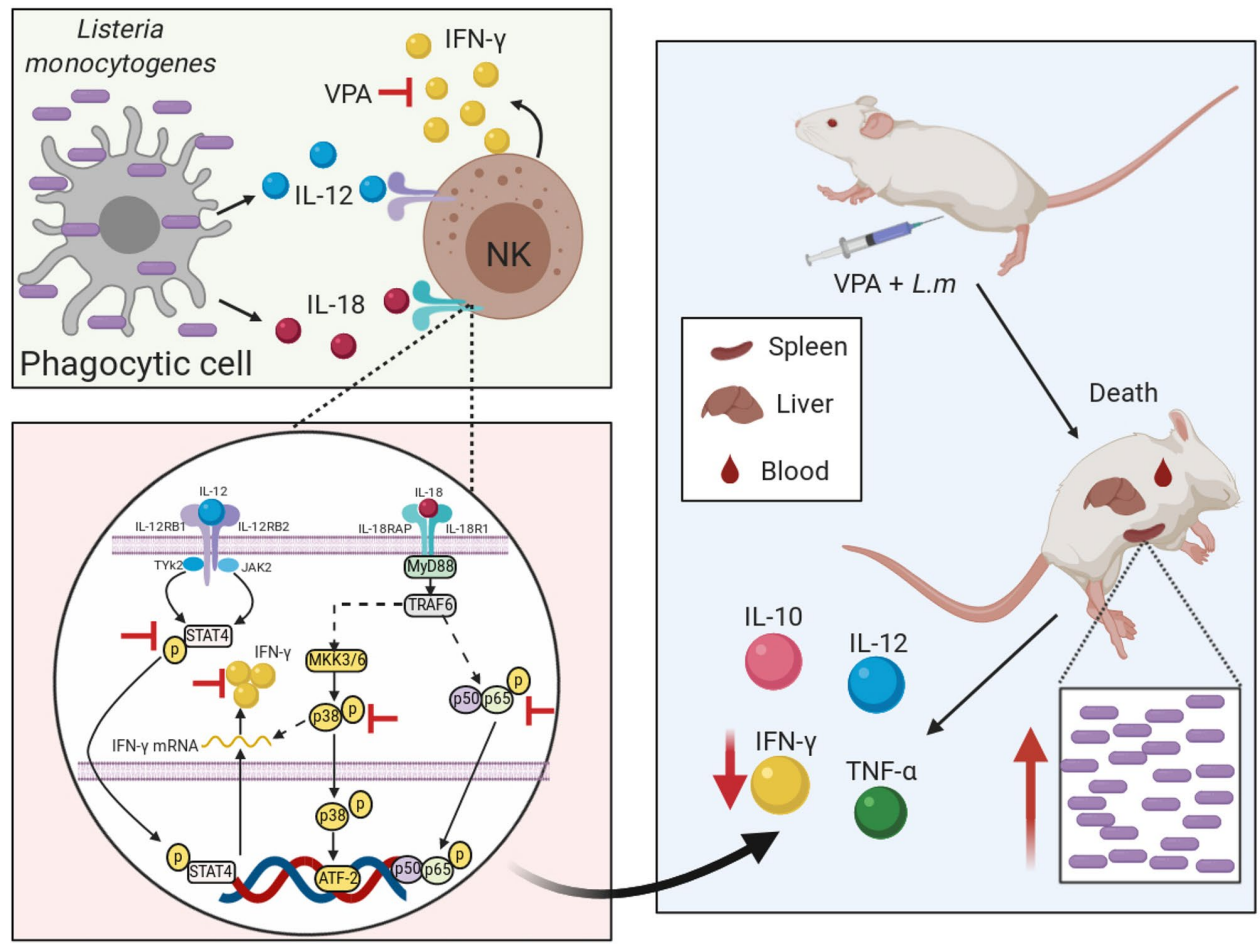
Valproic acid increases susceptibility to in vivo* Listeria monocytogenes* infection by inhibiting IFN-γ production and attenuates NK cell activation in vitro. VPA decreases IFN-γ production by NK cells in response to IL-12 and IL-18 produced by phagocytic cells in the context of L.m infection (upper-left panel). Without compromising the expression of IL-12 and IL-18 receptors, VPA inhibits the activation of STAT4, p38 and p65, all critical proteins downstream the IL-12 and IL-18 receptors signaling pathways (lower-left panel). The overall effect in vivo is a decreased level of IFN-γ in infected mice, promoting an increased bacterial load in spleen, liver and blood, and decreased resistance to L.m infection. Noteworthy, in this model VPA effect is cytokine specific as only IFN-γ expression, but not that of IL-10, IL-12 or TNF-α, was decreased (right panel). This figure was created with BioRender.com.

In conclusion, our study shows that VPA increases susceptibility to *Listeria monocytogenes* infection by decreasing IFN-γ production. In addition, our in vitro findings suggest that this effect may be partially explained by an impairment of the IL-12 and IL-18 signaling pathways required for IFN-γ production by NK cells during L.m infection (Fig. 8).

## Methods

### Mice

Female BALB/c mice aged 5–6 weeks with a weight of 18–19 g were provided by UPEAL, UAM, Mexico. All animals were housed under standard conditions and had access to water and food ad libitum. All experiments were reviewed and approved by the Research Ethics Committee of the ENCB, IPN. (ZOO-017-2019) in accordance to Mexican regulations.

### Ethical approval

All methods were carried out in accordance to Mexican relevant guidelines and regulations. All animal procedures were reviewed and approved by the Research Ethics Committee of the ENCB, IPN (ZOO-017–2019).

### Listeria monocytogenes

L.m strain 1778 + H 1b (ATCC 43249, USA, Manassas, VA, USA) was cultured in brain heart infusion broth (BHI, BD-Difco, USA) for 18 h at 37 °C with constant shaking. Cultures were washed with Hanks Balanced Saline Solution (HBSS) (Life Technologies, USA) and bacterial pellets resuspended in RPMI-1640 GlutaMAX (Life Technologies, USA) supplemented with 40% Fetal Bovine Serum (Life Technologies, USA) and frozen at − 70 °C until use. Bacterial viability was determined after serial dilutions and seeding in BHI agar plates, which were incubated at 37 °C for 18–24 h.

### In vivo* infection*

All mice were randomly assigned to one of the following groups: (1) saline group, animals were administered sterile saline solution (s.s) (PiSA, Mexico); (2) VPA group, animals were administered valproic acid (VPA) (Depakene; Abbott, USA) at 500 mg/kg in s.s; (3) L.m group, animals were treated with s.s. and 15 min later infected with 2.5 × 10^5^ CFU L.m in s.s; (4) VPA + L.m group, animals were treated with VPA and 15 min later infected with L.m*.* VPA, s.s, and L.m were administered intraperitoneally (i.p). The dose and time of VPA administration were chosen based on a mouse model of sepsis induced by puncture and ligation of cecum (CLP)^69^, and an infection model by *Klebsiella pneumoniae* and *Candida albicans*^8^.

Body weight, mice survival, bacterial load, splenic index and serum cytokines were evaluated every 24 h. The survival curve was stopped at 144 h hours post-infection (hpi). Survival was reported as a percentage. Mice that lost more than 20% of body weight were euthanized to avoid unnecessary distress.

To determine the bacterial load, L.m group and L.m + VPA group were euthanized and then, the abdominal and thoracic cavity were opened. 100  μL of blood was extracted by cardiac puncture and diluted with 900 μL of sterile water for 5 min. Spleen and liver were removed, mechanically dissociated, and added sterile water up to a final volume of 5 mL. Cell suspensions were homogenized and serial decimal dilutions were prepared in s.s. 20 μL of each dilution were cultured by triplicate in BHI agar plates at 37 °C for 18–24 h. After incubation, the viable count of L.m was assessed. The data are expressed as CFU/organ or CFU/100 μL of blood.

Splenic index was calculated by evaluating the weight of mice at 24 and 48 h. After euthanization, spleen was extracted and weighed. Splenic index = [spleen weight (g)/mouse weight (g)] × 100.

For quantification of serum cytokines (IFN-γ, TNF-α, IL-12p70 and IL-10), 400 μL blood was extracted via submandibular vein from mice in each experimental group. Blood was collected in capillary blood collection tubes with separator gel (BD Microtainer, USA) and centrifuged to obtain serum. Cytokines were quantified by ELISA (Biolegend, USA) following manufacturer’s instructions.

### Bacterial growth curve

L.m was cultured in BHI broth overnight. 20 µL of this culture were added to tubes with BHI broth or BHI broth plus 52 mM VPA and incubated at 37 °C with constant shaking. Bacterial viability was determined at 0, 3, 18 and 24 h after serial dilutions and seeding on BHI agar plates, which were incubated at 37 °C for 18–24 h.

### Viability assay

1 × 10^6^ splenocytes of healthy BALB/c were treated with VPA at different concentrations (1, 2, 5 and 10 mM) for 24 h. Then cells were washed and stained with 1 μg/mL Annexin V (BioLegend, USA) and 0.5 μg/mL propidium iodide (eBioscience, USA). After staining, cell viability was measured by flow cytometry.

### In vitro* infection and IFN-*γ* production*

1 × 10^6^ splenocytes of healthy BALB/c mice were preincubated with or without 2 Mm VPA for 1 h, and then infected with L.m at a Multiplicity of Infection (MOI) of 0.1. After 24 h of culture, supernatants were collected and IFN-γ production was evaluated by ELISA.

For the evaluation of cell populations producing IFN-γ by flow cytometry, we followed a previously reported protocol^24^. Briefly, splenocytes were infected with L.m for 18 h. During the last 4 h of culture, Brefeldin A (a protein transport inhibitor) (Biolegend, USA) was added. Next, cells were washed, blocked with anti-CD16/32 (Mouse BD Fc Block, clone: 2.4G2. BD-Biosciences, USA), stained with a mixture of fluorochrome-conjugated antibodies to CD3 (clone: 145-2C11), CD4 (clone: GK1.5), CD8 (clone: 53-6.7), CD49b (clone: DX5), TCRγδ (clone: GL3), B220 (clone: RA3-6B2), all from Biolegend, USA; CD11b (clone: HL3, BD-Biosciences, USA). Cells were fixed, permeabilized. stained with anti-IFN-γ (clone; XMG1.2. Biolegend, USA) and analyzed by flow cytometry.

### NK cells isolation

NK cells were obtained from spleens of healthy BALB/c mice using an NK cell negative selection isolation kit II (Militenyi Biotec, USA). The enrichment was ≥ 90% as determined by flow cytometry after staining with antibodies to CD3 (clone: 145-2C11) and CD49b (clone: DX5).

### IFN-γ production by NK cells

5X10^4^ NK cells were cultured in the presence or absence of 2 mM VPA for 1 h and then 20 ng/mL of murine recombinant IL-12p70 (Peprotech, USA) and 100 ng/mL of murine recombinant IL-18 (R&D systems, USA) were added. After 18 h of culture, supernatants were collected for IFN-γ detection by ELISA.

### Expression of IL-12 and IL-18 receptors on NK cells

NK cells were treated or not with VPA for 19 h and the expression of IL-12 and IL-18 receptors was evaluated by flow cytometry, using biotinylated antibodies against IL-12Rβ2 (clone: REA200; Militenyi Biotec, USA) or anti-IL-18Rα (REA947; Militenyi Biotec, USA) and stained with streptavidin-APC (BD-Bioscience, USA), prior blocking with anti-CD16/32.

### STAT4, p65 (NF-κB) and p38 phosphorylation in NK cells

NK cells were cultured in the presence or absence of VPA for 1 h and then stimulated with IL-12p70 plus IL-18 for 15 min to evaluate p-STAT4, 30 min for p-p65 and 60 min for p-p38. Then, the cells were preserved with Fixation buffer (BD-Bioscience, USA) and permeabilized with 0.5 × Perm buffer IV (BD-Bioscience, USA). After blocking with anti-CD16/32, the cells were stained with antibodies to p-STAT4 (clone: 38/p-Stat4), p-p65 (clone: K10-895.12.50) or p-p38 (Clone: 36/p38). All antibodies were from BD-Biosciences, USA.

### Flow cytometry

All cell samples stained with fluorochrome-conjugated antibodies to cell surface markers, intracellular IFN-γ and intracellular phosphorylated proteins were acquired using FACSCalibur (BD Biosciences) and analyzed with FlowJo v6.0 software.

### Statistical analysis

All statistical analyses were performed with SigmaPlot software version 14.0, from Systat Software, Inc., San Jose California USA, www.systatsoftware.com. Data normality was assessed by Kolmogorov–Smirnov with Lilliefors correction. Data are shown as mean ± standard error mean (s.e.m) or median and range or adjusted means with 95% confidence intervals, as appropriate. For comparisons between two groups, Mann–Whitney rank sum test with Yates correction was used. For comparisons between two or more groups, Kruskal–Wallis test was used. For comparisons between two or more groups with two factors, two way-analysis of variance (ANOVA) with Student–Newman–Keuls (SNK) post-hoc was used. For comparisons between two or more groups with two factors and repeated measures, two way-repeated measures-ANOVA (RM-ANOVA) with SNK post-hoc was used. One way-Analysis of Covariance (ANCOVA) was used to evaluate variations in the weight of the animals. The Kaplan–Meier method was used for survival and differences were analyzed by the log-rank sum test. A value of p < 0.05 was considered to be significant.

## Supplementary information

Supplementary Information.

## Data Availability

All data generated or analyzed during this study are included in this published article (and its Supplementary Information files).

## References

[1] Chateauvieux S, Morceau F, Dicato M, Diederich M (2010). Molecular and therapeutic potential and toxicity of valproic acid. J. Biomed. Biotechnol..

[2] Monti B, Polazzi E, Contestabile A (2009). Biochemical, molecular and epigenetic mechanisms of valproic acid neuroprotection. Curr. Mol. Pharmacol..

[3] Duenas-Gonzalez A (2008). Valproic acid as epigenetic cancer drug: Preclinical, clinical and transcriptional effects on solid tumors. Cancer Treat. Rev..

[4] Falkenberg KJ, Johnstone RW (2014). Histone deacetylases and their inhibitors in cancer, neurological diseases and immune disorders. Nat. Rev. Drug Discov..

[5] Soria-Castro R (2019). Exploring the drug repurposing versatility of valproic acid as a multifunctional regulator of innate and adaptive immune cells. J. Immunol. Res..

[6] Mombelli M (2011). Histone deacetylase inhibitors impair antibacterial defenses of macrophages. J. Infect. Dis..

[7] Rodríguez-López GM (2020). The histone deacetylase inhibitor valproic acid attenuates phospholipase Cγ2 and IgE-mediated mast cell activation. J. Leukoc. Biol..

[8] Roger T (2011). Histone deacetylase inhibitors impair innate immune responses to Toll-like receptor agonists and to infection. Blood.

[9] Caldiroli E (1998). Peripheral benzodiazepine receptor expression on leukocytes and neutrophil function during anticonvulsant monotherapy. Pharmacology.

[10] Alvarez-Breckenridge CA (2012). The histone deacetylase inhibitor valproic acid lessens nk cell action against oncolytic virus-infected glioblastoma cells by inhibition of STAT5/T-BET signaling and generation of gamma interferon. J. Virol..

[11] Bhat J, Oberg HH, Kabelitz D (2015). Modulation of human gamma/delta T-cell activation and phenotype by histone deacetylase inhibitors. Cell. Immunol..

[12] White CA (2014). Histone deacetylase inhibitors upregulate B cell microRNAs that silence AID and Blimp-1 expression for epigenetic modulation of antibody and autoantibody responses. J. Immunol..

[13] Ersvaer E, Brenner AK, Vetås K, Reikvam H, Bruserud Ø (2015). Effects of cytarabine on activation of human T cells—cytarabine has concentration-dependent effects that are modulated both by valproic acid and all-trans retinoic acid. BMC Pharmacol. Toxicol..

[14] Zhang Z, Wu Y, Schluesener HJ (2012). Valproic acid ameliorates inflammation in experimental autoimmune encephalomyelitis rats. Neuroscience.

[15] Wongrakpanich S, Wongrakpanich A, Melhado K, Rangaswami J (2018). A comprehensive review of non-steroidal anti-inflammatory drug use in the elderly. Aging Dis..

[16] Spits H, Bernink JH, Lanier L (2016). NK cells and type 1 innate lymphoid cells: partners in host defense. Nat. Immunol..

[17] Kak G, Raza M, Tiwari BK (2018). Interferon-gamma (IFN-γ): exploring its implications in infectious diseases. Biomol. Concepts.

[18] Humann J, Lenz LL (2010). Activation of naive NK cells in response to listeria monocytogenes requires IL-18 and contact with infected dendritic cells. J. Immunol..

[19] Marçais A (2013). Regulation of mouse NK cell development and function by cytokines. Front. Immunol..

[20] Vázquez-boland JA (2001). *Listeria* pathogenesis and molecular virulence determinants *Listeria* Pathogenesis and molecular virulence determinants. Clin. Microbiol. Rev..

[21] Harty JT, Bevant MJ (1995). Specific immunity to listeria monocytogenes in the absence of IFNγ. Immunity.

[22] Buchmeier NA, Schreiber RD (1985). Requirement of endogenous interferon-gamma production for resolution of Listeria monocytogenes infection. Proc. Natl. Acad. Sci. U. S. A..

[23] D’Orazio SEF (2019). Innate and adaptive immune responses during Listeria monocytogenes Infection. Microbiol. Spectr..

[24] Thäle C, Kiderlen AF (2005). Sources of interferon-gamma (IFN-γ) in early immune response to Listeria monocytogenes. Immunobiology.

[25] Kubota K, Kadoya Y (2011). Innate IFN-g-producing cells in the spleen of mice early after Listeria monocytogenes infection: importance of microenvironment of the cells involved in the production of innate IFN-g. Front. Immunol..

[26] Nomura T (2002). Essential role of interleukin-12 (IL-12) and IL-18 for gamma interferon production induced by listeriolysin O in mouse spleen cells. Infect. Immun..

[27] Rossi LE (2012). Histone deacetylase inhibitors impair NK cell viability and effector functions through inhibition of activation and receptor expression. J. Leukoc. Biol..

[28] Bronte V, Pittet MJ (2013). The spleen in local and systemic regulation of immunity. Immunity.

[29] Tripp CS, Gately MK, Hakimi J, Ling P, Unanue ER (1994). Neutralization of IL-12 decreases resistance to Listeria in SCID and C.B-17 mice. Reversal by IFN-gamma. J. Immunol..

[30] Tripp CS, Wolf SF, Unanue ER (1993). Interleukin 12 and tumor necrosis factor alpha are costimulators of interferon gamma production by natural killer cells in severe combined immunodeficiency mice with listeriosis, and interleukin 10 is a physiologic antagonist. Proc. Natl. Acad. Sci. U. S. A..

[31] García VE (1999). IL-18 promotes type 1 cytokine production from NK cells and T cells in human intracellular infection. J. Immunol..

[32] Fagundes CT (2011). IFN-γ production depends on IL-12 and IL-18 combined action and mediates host resistance to dengue virus infection in a nitric oxide-dependent manner. PLoS Negl. Trop. Dis..

[33] Mavropoulos A, Sully G, Cope AP, Clark AR (2005). Stabilization of IFN-γ mRNA by MAPK p38 in IL-12- and IL-18-stimulated human NK cells. Blood.

[34] Vivier E, Tomasello E, Baratin M, Walzer T, Ugolini S (2008). Functions of natural killer cells. Nat. Immunol..

[35] Paolini R, Bernardini G, Molfetta R, Santoni A (2015). NK cells and interferons. Cytokine Growth Factor Rev..

[36] Nieto-Patlán E (2019). Valproic acid promotes a decrease in mycobacterial survival by enhancing nitric oxide production in macrophages stimulated with IFN-γ. Tuberculosis.

[37] Amirzargar MA (2017). Anti-inflammatory effects of valproic acid in a rat model of renal ischemia/reperfusion injury: alteration in cytokine profile. Inflammation.

[38] Ji M (2013). Valproic acid attenuates lipopolysaccharide-induced acute lung injury in mice. Inflammation.

[39] Wu J (2013). Class i histone deacetylase inhibitor valproic acid reverses cognitive deficits in a mouse model of septic encephalopathy. Neurochem. Res..

[40] Teixeira HC, Kaufmann SHE (1994). Role of NK1.1+ cells in experimental listeriosis: NK1+ cells are early IFN-γ producers but impair resistance to Listeria monocytogenes infection. J. Immunol..

[41] Takada H, Matsuzaki G, Hiromatsu K, Nomoto K (1994). Analysis of the role of natural killer cells in Listeria monocytogenes infection: relation between natural killer cells and T-cell receptor gamma delta T cells in the host defence mechanism at the early stage of infection. Immunology.

[42] Clark SE (2016). Bacterial manipulation of NK cell regulatory activity increases susceptibility to listeria monocytogenes infection. PLoS Pathog..

[43] Berg RE, Crossley E, Murray S, Forman J (2003). Memory CD8+ T cells provide innate immune protection against listeria monocytogenes in the absence of cognate antigen. J. Exp. Med..

[44] Berg RE, Crossley E, Murray S, Forman J (2005). Relative contributions of NK and CD8 T cells to IFN-γ mediated innate immune protection against listeria monocytogenes. J. Immunol..

[45] Santini V, Gozzini A, Ferrari G (2007). Histone deacetylase inhibitors: molecular and biological activity as a premise to clinical application. Curr. Drug Metab..

[46] Dulson SJ, Watkins EE, Crossman DK, Harrington LE (2019). STAT4 directs a protective innate lymphoid cell response to gastrointestinal infection. J. Immunol..

[47] Strengell M (2003). IL-21 in synergy with IL-15 or IL-18 enhances IFN-γ production in human NK and T cells. J. Immunol..

[48] Wieczorek M, Ginter T, Brand P, Heinzel T, Krämer OH (2012). Acetylation modulates the STAT signaling code. Cytokine Growth Factor Rev..

[49] Ogbomo H, Michaelis M, Kreuter J, Doerr HW, Cinatl J (2007). Histone deacetylase inhibitors suppress natural killer cell cytolytic activity. FEBS Lett..

[50] Ichiyama T (2000). Sodium valproate inhibits production of TNF-α and IL-6 and activation of NF-κB. Brain Res..

[51] Jambalganiin U (2014). A novel mechanism for inhibition of lipopolysaccharide-induced proinflammatory cytokine production by valproic acid. Int. Immunopharmacol..

[52] Nencioni A (2007). Histone deacetylase inhibitors affect dendritic cell differentiation and immunogenicity. Clin. Cancer Res..

[53] Seet LF, Toh LZ, Finger SN, Chu SWL, Wong TT (2019). Valproic acid exerts specific cellular and molecular anti-inflammatory effects in post-operative conjunctiva. J. Mol. Med..

[54] Gao Q (2020). Sodium valproate attenuates the iE-DAP induced inflammatory response by inhibiting the NOD1-NF-κB pathway and histone modifications in bovine mammary epithelial cells. Int. Immunopharmacol..

[55] Adhya D, Dutta K, Kundu K, Basu A (2013). Histone deacetylase inhibition by Japanese encephalitis virus in monocyte/macrophages: a novel viral immune evasion strategy. Immunobiology.

[56] Xie N (2010). The role of p38 MAPK in valproic acid induced microglia apoptosis. Neurosci. Lett..

[57] Leong IL (2018). Valproic acid inhibits ATP-triggered Ca2+ release via a p38-dependent mechanism in bEND.3 endothelial cells. Fundam. Clin. Pharmacol..

[58] Anand R, Kothary PC, Del Monte MA (2018). Valproic acid inhibits human retinal pigment epithelial (hRPE) cell proliferation via a P38 MAPK signaling mechanism. Adv. Exp. Med. Biol..

[59] Kim K (2012). Effect of valproic acid on acute lung injury in a rodent model of intestinal ischemia reperfusion. Resuscitation.

[60] Long J (2015). Valproic acid ameliorates graft-versus-host disease by downregulating Th1 and Th17 cells. J. Immunol..

[61] Brauner H (2010). Distinct phenotype and function of NK cells in the pancreas of nonobese diabetic mice. J. Immunol..

[62] Batista MD (2013). Skewed distribution of natural killer cells in psoriasis skin lesions. Exp. Dermatol..

[63] Weber B (2017). Distinct interferon-gamma and interleukin-9 expression in cutaneous and oral lichen planus. J. Eur. Acad. Dermatol. Venereol..

[64] Chalan P, Bijzet J, Kroesen BJ, Boots AMH, Brouwer E (2016). Altered natural killer cell subsets in seropositive arthralgia and early rheumatoid arthritis are associated with autoantibody status. J. Rheumatol..

[65] Krämer B (2013). Role of the NK cell-activating receptor CRACC in periodontitis. Infect. Immun..

[66] Suzuki M (2017). The cellular and molecular determinants of emphysematous destruction in COPD. Sci. Rep..

[67] Tenger C, Sundborger A, Jawien J, Zhou X (2005). IL-18 accelerates atherosclerosis accompanied by elevation of IFN-γ and CXCL16 expression independently of T cells. Arterioscler. Thromb. Vasc. Biol..

[68] Drevets DA, Bronze MS (2008). Listeria monocytogenes: epidemiology, human disease, and mechanisms of brain invasion. FEMS Immunol. Med. Microbiol..

[69] Takebe M (2014). Inhibition of histone deacetylases protects septic mice from lung and splenic apoptosis. J. Surg. Res..

