# Supplementary material for: Valproic acid inhibits interferon-γ production by NK cells and increases susceptibility to Listeria monocytogenes infection
Source: Sci Rep. 2020 Oct 20;10:17802. doi: 10.1038/s41598-020-74836-w (PMC7576816; doi:10.1038/s41598-020-74836-w)
Supplement: Supplementary file 1 — Supplementary Information. [file 41598_2020_74836_MOESM1_ESM.pdf]

Valproic acid inhibits interferon-γ production by NK cells and increases susceptibility to *Listeria monocytogenes* infection

Rodolfo Soria-Castro[1], Alma D. Chávez-Blanco[2], Blanca Estela García-Pérez[1,3], Isabel Wong-Baeza[1], Raúl Flores-Mejía[4], Fabián Flores-Borja[5], Sergio Estrada-Parra[1], Iris Estrada-García[1], Jeanet Serafín-López[1]*, Rommel Chacón-Salinas[1]*.

1. Departamento de Inmunología, Escuela Nacional de Ciencias Biológicas, Instituto Politécnico Nacional (ENCB-IPN), Mexico City, Mexico.

2. Subdirección de Investigación Básica, Instituto Nacional de Cancerología (INCan), México City, Mexico.

3. Departamento de Microbiología, Escuela Nacional de Ciencias Biológicas, Instituto Politécnico Nacional (ENCB-IPN), Mexico City, Mexico.

4. Laboratorio 103, Sección de Estudios de Posgrado e Investigación, Escuela Superior de Medicina, Instituto Politécnico Nacional (ESM-IPN), Mexico City, Mexico.

5. Centre for Immunobiology and Regenerative Medicine. Barts & The London School of Medicine and Dentistry. Queen Mary University of London. London, United Kingdom.

* Corresponding authors at:

Departamento de Inmunología, Escuela Nacional de Ciencias Biológicas, Instituto Politécnico Nacional, Carpio y Plan de Ayala s/n Col. Santo Tomás, Mexico city. C.P. 11340, México.

Phone:+525557296300 ext. 62507

E-mail addresses: jeaserafin@hotmail.com (J. Serafín-Lopez),

rommelchacons@yahoo.com.mx, rchacons@ipn.mx (R. Chacón-Salinas)

Supplementary figures

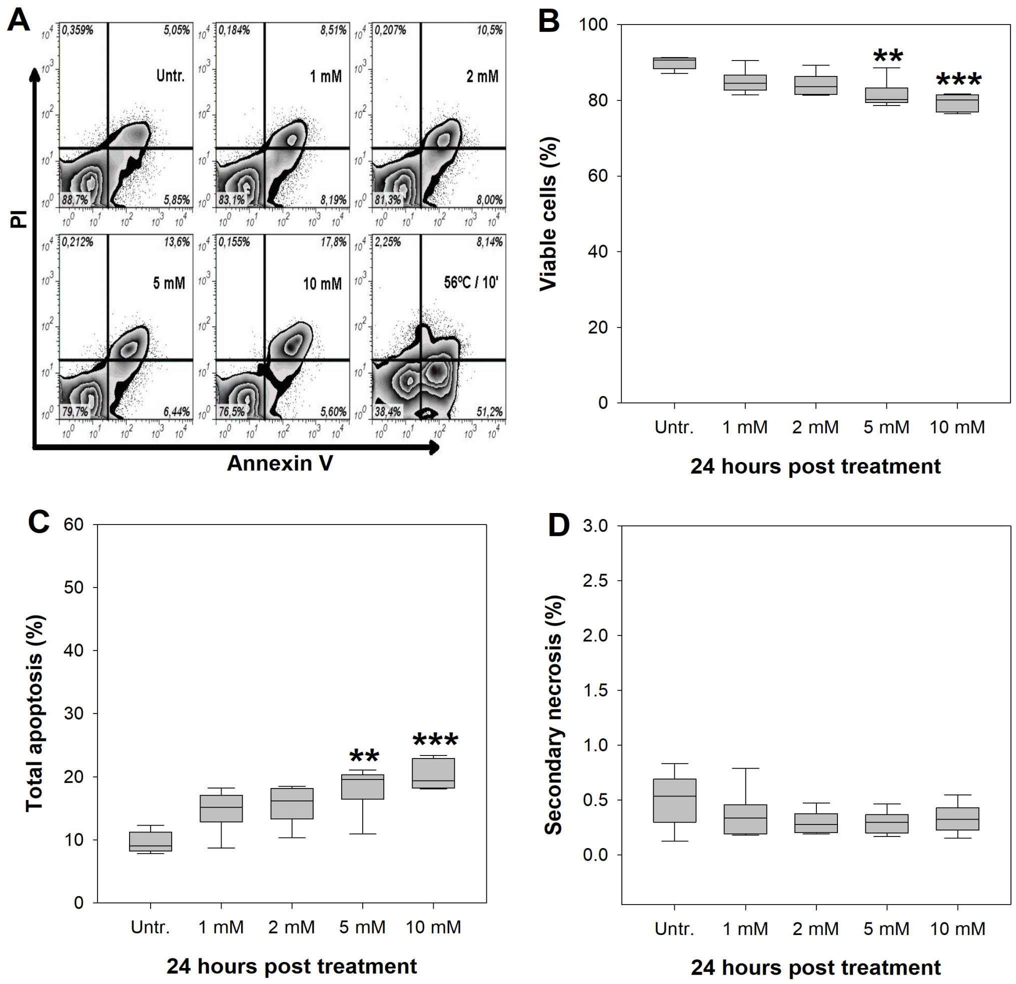

Supplementary Figure S1. Evaluation of the effect of valproic acid on the viability of spleen cells. $1X10^6$ Spleen cells from BALB/c mice were treated with different doses of VPA for 24 h. Cell viability was determined with Annexin V and propidium iodide (PI) staining and measured by flow cytometry. (A) Representative zebra-plots of splenocytes staining with Annexin V and PI. (B) Percentage of viable cells after VPA treatment (Annexin V-/PI-). (C) Percentage of total apoptotic cells represented as the sum of the percentage of early apoptotic cells (Annexin V+/PI-) and the percentage of late apoptotic cells (Annexin V+/PI+). (D) Percentage of necrotic cells (Annexin V-/PI-). (n=6 per group; **P<0.01, ***P<0.001). Data are expressed as median and range; Kruskal-Wallis.

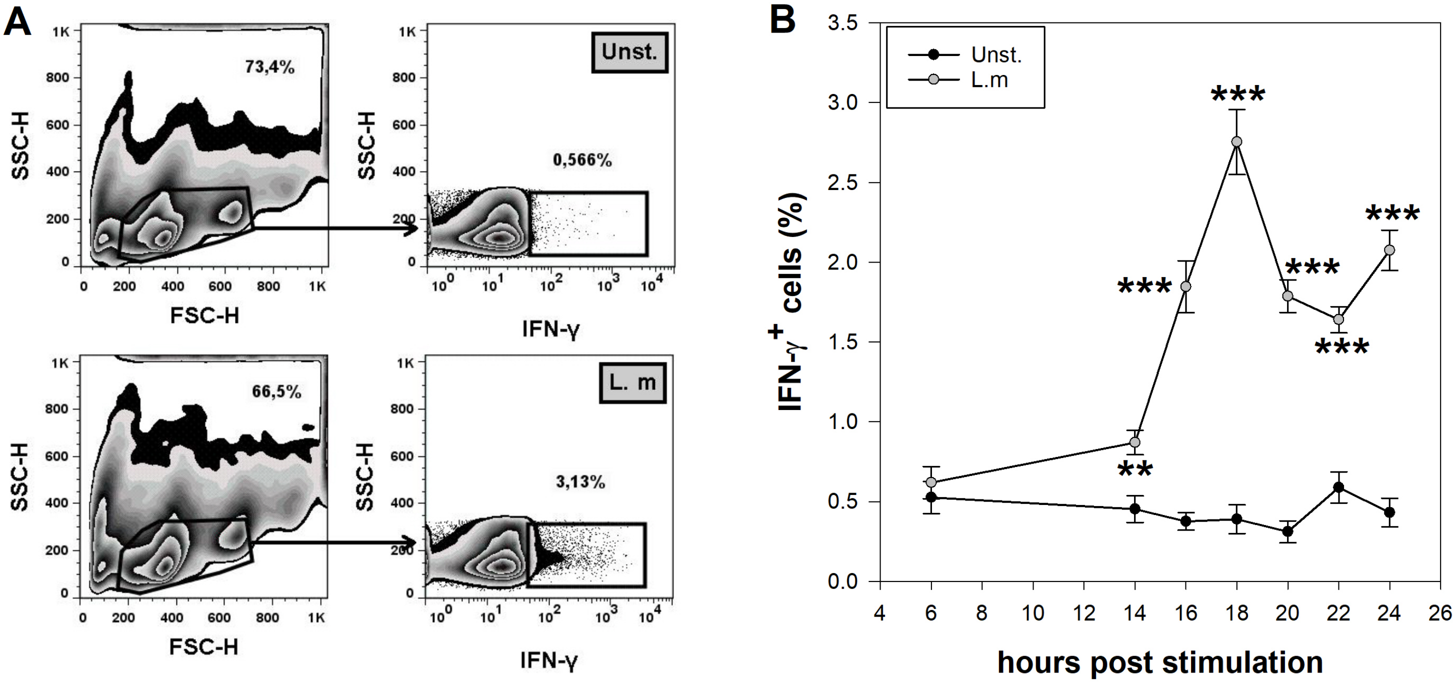

Supplementary Figure S2. Kinetics of IFN-γ-producing lymphocytes in response to *Listeria monocytogenes* infection *in vitro*. 1X10^6 splenocytes were infected with L.m and the percentage of IFN-γ-producing lymphocytes was evaluated at different time points by flow cytometry. (A) Representative zebra plots showing the frequency of IFN-γ+ lymphocytes in uninfected and L.m-infected cultures. (B) Kinetics of total IFN-γ-producing lymphocytes (n=3 per group; **P<0.01, 6 h vs 14 h and ***P<0.001, 6 h vs 16, 18, 20, 22 and 24 h in L.m group). Data are expressed as mean ± s.e.m; two way-RM ANOVA.

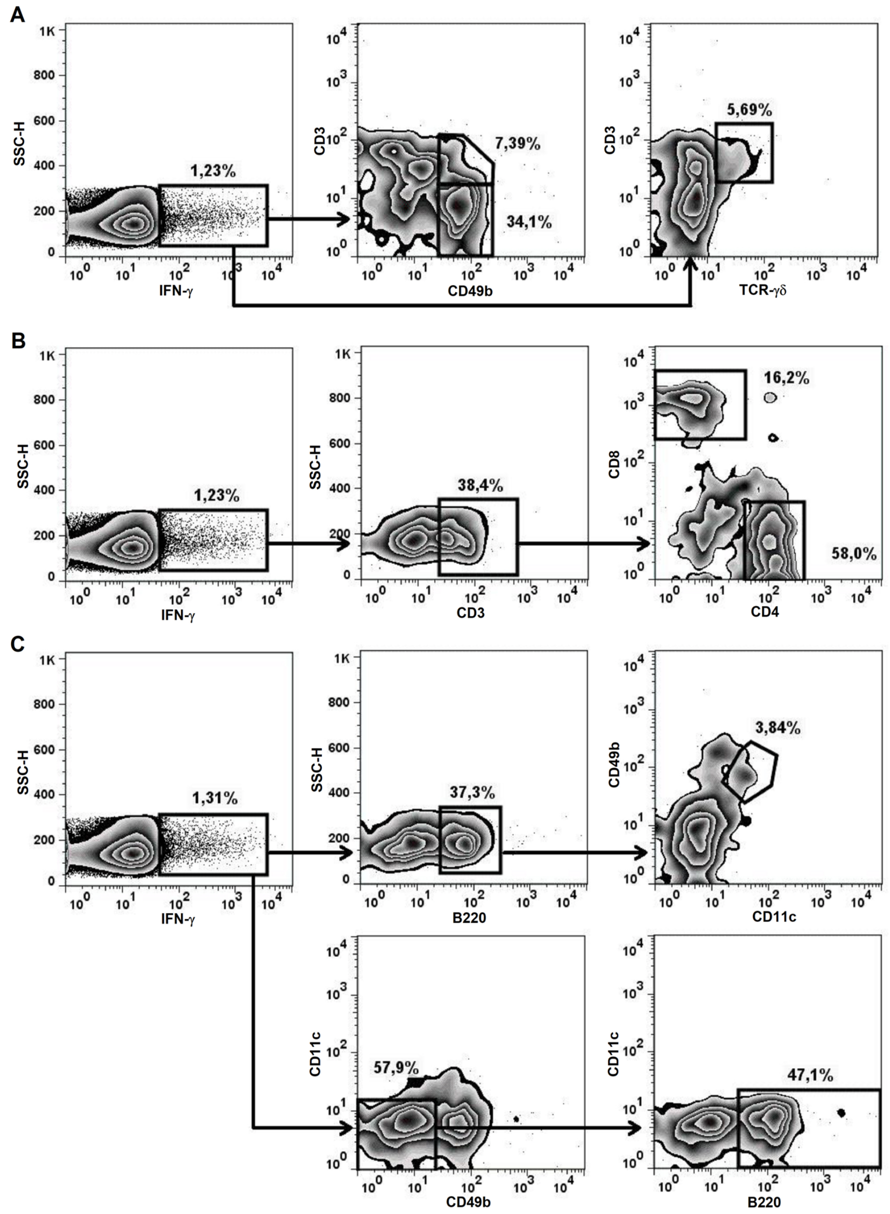

Supplementary Figure S3. Identification of IFN-γ-producing splenocyte cell populations during *Listeria monocytogenes in vitro* infection. $1X10^6$ splenocytes were infected with L.m for 18h and the percentage of the different IFN-γ-producing cells was evaluated by flow cytometry. (A) Representative zebra plots showing the percentage of NK (CD3- CD49b+), NKT (CD3+ CD49b+) and γδ T cells (CD3+ TCRγδ); (B) CD3+CD4+ and CD3+CD8+ T cells; and (C) IKDC (B220+ CD11clo CD49b+) and B cells (CD11c- CD49b- B220+) within total IFN-γ-producing lymphocytes.

Supplementary Table S1. Total number of IFN-γ producing cells in BALB/C spleen during *Listeria monocytogenes* infection *in vitro*.

IFN-γ^+ cells/1x10^6 splenocytes	Uninfected	*Listeria moncytogenes*	
	Median and (25th-75th percentile)	Median and (25th-75th percentile)	*P value
NK cells	62.5 (45-86.25)	2782.5 (1727.5-3342.5)	0.002
NKT cells	110 (65-131.25)	435 (306.25-682.5)	0.002
Tγδ cells	147.5 (86.25-216.25)	317.5 (201.25-508.75)	0.015
CD4 T cells	600 (317.5-687.5)	1105 (707.5-2057.5)	0.041
CD8 T cells	127.5 (98.75-233.75)	212.5 (112.5-400)	0.394
IKDC	35 (30-56.25)	45 (25-111.25)	0.485
B cells	1492.5 (1370-2140)	1220 (1043.75-3337.5)	0.589

*** U Mann-Whitney with Yates correction**
